# Use of CytoSorb® hemoadsorption column during prolonged cardiopulmonary bypass in complex cardiac surgery patient

**DOI:** 10.1186/s13019-022-01922-7

**Published:** 2022-07-07

**Authors:** Marianne Alarie, Maggie Savelberg, Danika Vautour, Igo B. Ribeiro

**Affiliations:** Kingston Health Sciences Centre, Kingston, ON Canada

**Keywords:** CytoSorb, Bypass, Hemoadsorption, Cytokines, Inflammation, Case report

## Abstract

**Background:**

Complex cardiac surgery and prolonged cardiopulmonary bypass are associated with significant activation of the systemic inflammatory response system. Pro-inflammatory cytokines, oxygen free radicals and complement activation products contribute to postoperative complications and multiorgan injury. CytoSorb® hemoadsorption therapy has been suggested to alleviate the hyperinflammatory response triggered by cardiopulmonary bypass during cardiac surgery.

**Case presentation:**

We describe the use of CytoSorb® hemoadsorption therapy in a 61-year-old male presenting for aortic valve replacement, mitral valve replacement, tricuspid valve repair, coronary artery bypass grafting and left atrial appendage clip.

**Conclusion:**

We were able to demonstrate that CytoSorb® use during cardiopulmonary bypass may be a safe and feasible adjunct therapy that may contribute to improved postoperative outcomes in a patient with complex cardiac disease.

## Background

Cardiopulmonary bypass (CPB) is routinely used to provide patient support during cardiac surgery. However, its use is associated with the activation of the systemic inflammatory response system [1, 2]. Contact of blood with the artificial extracorporeal surface, surgical trauma and ischemia all trigger the release of numerous inflammatory mediators. Proinflammatory cytokines (IL-6, IL-8, IL-10, TNF-a, etc.), oxygen free radicals and complement system activation products (C3a, C5a) contribute to postoperative complications such as hypotension via vasoplegia, bleeding, myocardial dysfunction and multiorgan injury [2, 3]. Mitigating the adverse effects that result from activation of the inflammatory response during cardiopulmonary bypass and cardiac surgery would be beneficial.

A novel hemoadsorption therapy, called CytoSorb® (CytoSorbents Corporation, Monmouth Junction, NJ, USA), has recently gained interest for its use in reducing excessive amounts of inflammatory mediators and alleviating the hyperinflammatory response in critically ill patients [3, 4]. It consists of a biocompatible porous polymer sorbent technology with hydrophobic beads that bind to a wide range of inflammatory mediators. The cartridge can be easily integrated into the CPB circuit [5]. Cytosorb® is currently approved in the European Union (EU) as an extracorporeal cytokine adsorber designed to mitigate the effects of the ‘cytokine storm’ that may result in inflammation and organ dysfunction [6]. CytoSorb® has also been granted the Food and Drug Administration (FDA) Breakthrough Designation approval for the removal of ticagrelor in cardiopulmonary bypass in cases of emergent cardiac surgery [7]. Studies show that the therapy is a safe and effective method of reducing bleeding complications in these patients [8]. Health Canada has granted approval for its use in hospitalized COVID-19 patients, but has yet to approve its use in cardiac surgery [7]. Indications for its use in the cardiac operating room include septic shock, vasoplegic shock, ticagrelor removal, endocarditis with valve surgeries, complex combined and/or redo surgeries, procedures with expected prolonged CPB times and patients with concomitant renal and/or liver dysfunction [6, 9]. Although scarce, studies have suggested the treatment is well tolerated and safe, and a promising therapeutic option for patients undergoing extracorporeal life support therapy [10–13].

Here, we present a case report of the use of CytoSorb® adjunct therapy in a patient who presented to our center with complex cardiac disease and multiple comorbidities. To our knowledge, this is the first case report describing its use in Canada, which may help guide the approval process for its use in cardiac surgery in the future.

## Case presentation

A 61-year-old male was assessed by cardiac surgery in consideration for mitral valve surgery following a congestive heart failure presentation. Transthoracic echocardiography showed significant mitral valve calcification resulting in severe mitral regurgitation and moderate mitral stenosis. The right and left ventricle had mild dysfunctions with an ejection fraction of 46%. Further evaluation with transesophageal echocardiography revealed moderate aortic cusp calcification with moderate stenosis. Moderate tricuspid regurgitation was also noted. Coronary angiography revealed the presence of significant coronary artery disease with second diagonal ostial stenosis.

The patient’s medical history includes end-stage renal disease requiring intermittent hemodialysis, autoimmune cytopenia (severe thrombocytopenia, neutropenia) with mild responsiveness to preoperative steroids, New York Heart Association class III heart failure, hypertension, chronic obstructive pulmonary disease, severe untreated sleep apnea, previous Grave’s disease diagnosis and atrial fibrillation. The patients’ preoperative blood work included a platelet count of 74 × 10^9^/L, hemoglobin of 91 g/L and hematocrit of 29%. Preoperative creatinine was 598 umol/L and glomerular filtration rate (GFR) was 8 mL/min/1.73 m2.

**Fig. 1:**
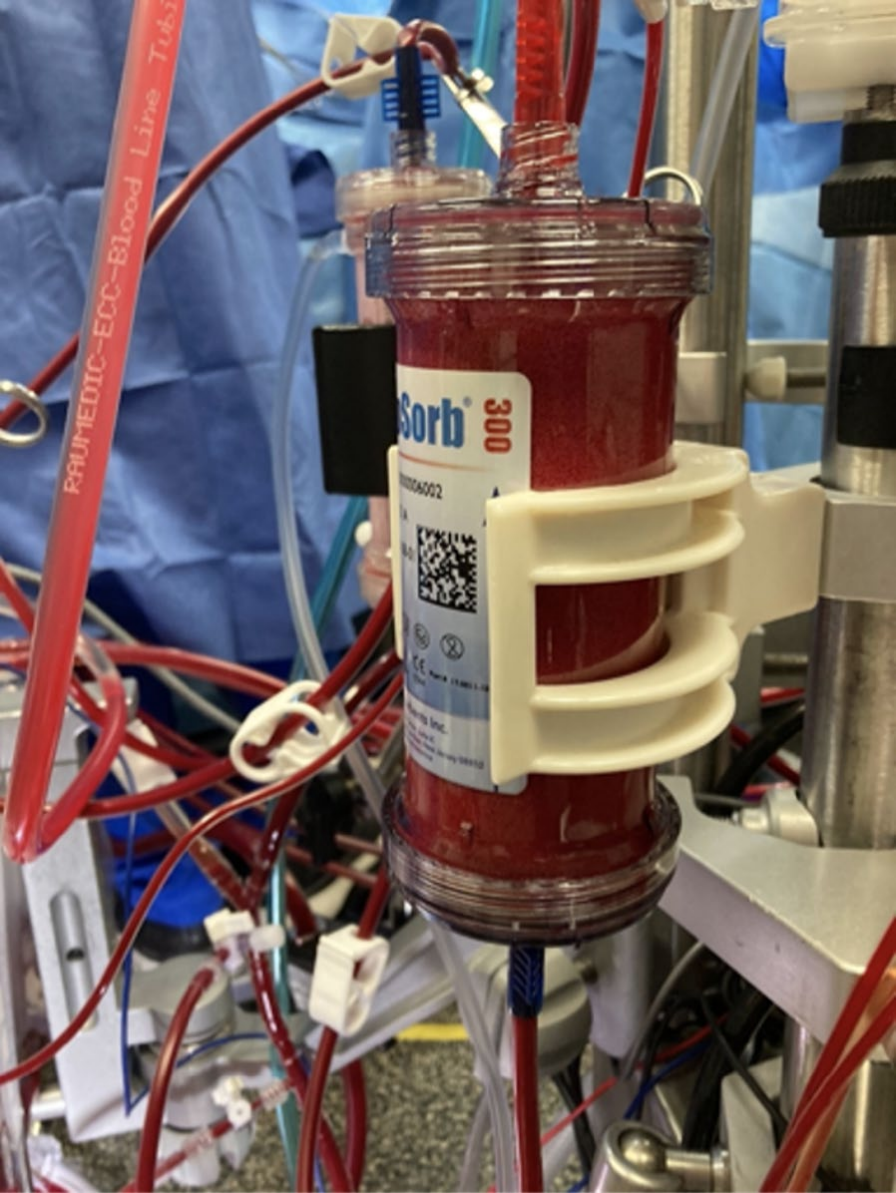
CytoSorb® in use

The patient had mitral valve replacement, aortic valve replacement, tricuspid valve repair, single coronary artery bypass grafting and left atrial appendage clip. Given the complexity of the surgery, the anticipated prolonged length of cardiopulmonary bypass, the associated risk of significant vasoplegia and the preoperative kidney dysfunction, a decision was made to integrate the CytoSorb® cartridge into the cardiopulmonary bypass circuit. The device was inserted between the recirculation line (high-pressure line) and the venous reservoir of the CPB circuit. The cartridge was placed in a parallel fashion with the hemoconcentrator (Fig. 1). The cartridge was primed and flushed with one liter of Ringer’s Lactate.

Following induction of anesthesia and prior to commencement of bypass, the patient required norepinephrine at 2 mcg/min for vasoactive support. Cardiopulmonary bypass was initiated and flow through the CytoSorb® began shortly after. Blood flow through the CytoSorb® was maintained from initiation of bypass until weaning, at which point the flow was stopped. Blood flow rates through the CytoSorb® were estimated to be between 200 and 230 mL/min. Anticoagulation was achieved using heparin with target activated clotting times (ACT) greater than 400 s, monitored every 20 to 30 min. Roughly 10 min into the bypass run, the patient required further vasoactive support and the norepinephrine infusion was increased to 5 mcg/min. The cross-clamp was applied and cardioplegia was administered using the Quest Myocardial Protection System™ 3 via retrograde and antegrade cardioplegia catheters. Myocardial quiescence was achieved in 62 s, with a total initial cardioplegia dose of 1480 mL. Cardioplegia was re-dosed every 10 to 15 min thereafter. Mean arterial blood pressures (MAP) were sustained around 45–50 mmHg with 5 mcg/min of norepinephrine for the first half hour of the bypass run. At the 40 min mark, MAP increased to a mean of 65–70 mmHg and the norepinephrine dose was reduced to 2 mcg/min. After one hour on bypass, MAP increased to 70–75 mmHg and norepinephrine was discontinued. Pressures above 60 mmHg were sustained for the remainder of the bypass run. Phenylephrine was bolused as required throughout the duration of bypass; however, additional need for vasoactive support was not required. Zero balance ultrafiltration was performed during bypass, with a total of 2.2 L of PrismaSol ‘0’ dialysate solution administered and 5.2 L of fluid removed via the hemoconcentrator. A total of 12.7 L of microplegia was delivered to ensure adequate myocardial protection.

The left atrial appendage was clipped first, using a #40 Gillinov-Cosgrove clip. A saphenous vein graft was then used to anastomose the second diagonal, after which the left atrium was opened to expose the mitral valve with a Cosgrove retractor. A cord-sparing technique was used to replace the mitral valve with a #29 Mosaic bioprosthesis. The left atrium was closed and the ascending aorta opened. A #25 Intuity valve was inflated in the standard fashion to replace the aortic valve. The ascending aorta was closed and the tricuspid valve was repaired with a #30 contour 3D ring under beating heart. The left atrium was then closed. Two ventricular and two atrial wires were inserted for epicardial pacing. In preparation for weaning from cardiopulmonary bypass, dobutamine and epinephrine drips were started at 7.5 mcg/kg/min and 5 mcg/min, respectively. Termination of bypass was uneventful. In an effort not to overload the right ventricle, leftover blood from the cardiopulmonary bypass circuit, including that of the CytoSorb®, was processed in a cell salvage device to be later transfused. Total bypass time was 154 min, with a cross-clamp time of 115 min. The patient required dobutamine 5 mcg/kg/min, norepinephrine 4 mcg/min, epinephrine 5 mcg/min and vasopressin 0.04 units/min for support 10 min post-bypass. Rotational thromboelastometry was performed to guide transfusion requirements. A total of 3 units of packed red blood cells (pRBC), 2 units of platelets and 1 unit of prothrombin complex concentrate were administered to treat perioperative anemia and coagulopathy. The patient was transferred to the cardiac intensive care unit on 5mcg/kg/min of dobutamine, 6 mcg/min of norepinephrine, 5mcg/min of epinephrine and 0.04 units/min of vasopressin. No adverse or any device-related side effects were documented during or after CytoSorb® treatment.

Dobutamine was discontinued within the first postoperative hour. Vasopressin was stopped 24 h postoperatively. By the end of the second postoperative day, norepinephrine drips had also been discontinued; however, the patient still required epinephrine at 4 mcg/min for vasoactive support (Table 1). Epinephrine was weaned and eventually discontinued 48 h postoperatively. Early postoperative blood work showed somewhat improved kidney function, with a creatinine of 394 umol/L and a GFR of 13 mL/min/1.73 m2 (Table 2). Lactate levels peaked at 3.4 mmol/L on the 6th postoperative hour and quickly normalized over the next 24 h. Following the first postoperative day, the patient had received an additional 3 units of pRBC, 2 units of plasma and 1 unit of platelets. Total chest tube drainage was measured to be 1080 mL and surgical drains were removed on the second postoperative day. The patient was extubated 48 h post-surgery. Hemodialysis treatments were resumed on the third postoperative day. The patient was transferred to the ward on the 6th postoperative day and discharged on the 11th postoperative day.

**Table 1:** Required vasoactive support

	Pre-CPB Post Anesthesia	During CPB	At CPB Wean	Leaving OR	6 h post surgery	24 h post surgery	48 h post surgery	72 h post surgery
Norepinephrine (mcg/min)	2	5	0	6	2	2	0	0
		*Off after 1 h						
Epinephrine (mcg/min)	0	0	5	5	4	4	2	0
Vasopressin (units/min)	0	0	0	0.04	0.04	0	0	0
Dobutamine (mcg/kg/min)	0	0	7.5	5	0	0	0	0

CPB, cardiopulmonary bypass; OR, operating room; h, hours

**Table 2:** Postoperative blood work

	Pre-CPB	Post surgery	6 h post surgery	24 h post surgery	48 h post surgery	72 h post surgery
Creatinine (umol/L)	598	389	412	464	357	470
GFR (mL/min/1.73 m2)	8	13	13	11	15	11
PLT × 10^9^/L	74	100	127	83	54	69
Hgb (g/L)	91	102	74	77	85	90
Hct (%)	29	31	23	23	25	27
Lactate (mmol/L)	0.6	1.3	3.4	1.1	–	–

CPB, cardiopulmonary bypass; h, hours; GFR, glomerular filtration rate; PLT, platelets; Hgb, hemoglobin; Hct, hematocrit

## Discussion

CytoSorb® is a 300 mL extracorporeal cytokine filter made of biocompatible polymer beads that can remove molecules between 10 and 50 kD [14]. Its goal is to reduce concentrations of proinflammatory cytokines while keeping the immune system intact. It is known that the use of cardiopulmonary bypass has an unpredictable contribution to the activation of the inflammatory response. Shear stress on blood elements, CPB-induced hypothermia, and blood exposure to artificial surfaces all contribute to the generation of perioperative systemic inflammatory response syndrome [15].

Although intraoperative use of the CytoSorb® adsorber has been suggested to decrease proinflammatory cytokines and improve post-surgical outcomes in cardiac surgery patients, little research exists demonstrating these effects. Bernardi et al. studied the effects of the novel adsorbent therapy in 37 patients undergoing elective cardiac surgery [11]. They measured differences in cytokine levels (IL-1β, IL-6, IL-18, TNF-α, and IL-10) within the first five postoperative days. Fluid components, blood products and catecholamine treatment were also analyzed. In comparison to the 18 controls, the 19 patients receiving CytoSorb® therapy did not show any reduction in their pro-inflammatory response, nor any changes in their perioperative course [11]. No differences in catecholamine requirements, postoperative fluid balances or blood administration were observed between patients who received CytoSorb® therapy and those who did not. Of note, however, they found that IL-10 showed a longer-lasting anti-inflammatory effect, which required further investigation. Similarly, Poli et al. measured the difference in key cytokine levels (IL-1a, IL-1b, IL-2, IL-4, IL-5, IL-6, IL-10, TNF-α, IFN-γ, MCP-1) in 30 patients undergoing elective cardiac surgery and deemed at risk of complications [10]. Cytokine levels were measured at anesthesia induction, at the end of CPB, as well as 6 and 24 h post-CPB initiation. They found that the intervention was not associated with a decrease in pro-inflammatory cytokine levels, nor any improvement in clinical outcomes [10]. No differences were found in terms of need for vasoconstrictors, postoperative acute kidney injury, and length of stay in the intensive care unit [10]. Conversely, Garau et al. found a reduction in pro-inflammatory cytokine levels of IL-8 and TNF-α in patients receiving CytoSorb® hemoadsorption therapy on CPB [16]. Cytokine levels were assessed prior to CPB, at the end of CPB, and 6 and 24 h post-CPB in 40 patients undergoing elective on-pump cardiac surgery. Although the study showed a reduction in some pro-inflammatory cytokines using hemoadsorption therapy, the differences in cytokine levels and cardiac-index between those treated with CytoSorb® therapy and those who were not were found to be minor and of short duration [16].

We did not measure cytokine levels before and after CytoSorb® use, and therefore cannot comment with regards to its impact on our patient’s inflammatory response. However, we felt that CytoSorb® therapy may have contributed to a decreased need for vasoactive support during and after surgery. Considering the length of time spent on cardiopulmonary bypass (154 min) and the complexity of a triple valve surgery in a critically ill patient, we considered this individual to be at high risk for vasoplegia following termination of cardiopulmonary bypass. In a retrospective case study of 40 patients presenting with multiorgan failure who received CytoSorb® therapy, Calabrò et al. observed a significant reduction of the vasoactive-inotropic score after 48 h of treatment as compared to baseline [17]. Of these patients, 19 underwent extracorporeal membrane oxygenation, 11 had an intra-aortic balloon pump, 9 had an Impella, 6 had a ventricular assist device and 18 were treated with continuous veno-venous hemofiltration. In these patients, CytoSorb® therapy was applied for an average length of 3 days. They found that CytoSorb® hemoadsorption helped decrease vasopressor requirements [17]. Due to a shorter treatment time, the question remains whether the same decrease in vasopressor requirements can be observed in patients receiving CytoSorb® therapy intraoperatively via the CPB circuit. In our patient, norepinephrine was required during the first hour of bypass, but was quickly discontinued. Further vasoactive support was not required until weaning. Despite the need for vasopressin until the 6th postoperative hour, small dose norepinephrine throughout the first postoperative day and epinephrine 48 h post-surgery, we felt that the patient required significantly less vasoactive support than originally anticipated. However, little evidence exists demonstrating these effects in cardiac surgery patients receiving hemadsorption therapy intraoperatively. Thus, further research will be required to determine whether integration of the cytokine adsorber into the CPB circuit during cardiac surgery results in significant reductions in vasopressor requirements.

CytoSorb® use is contraindicated in patients with platelet counts less than 20 × 10^9^/L [5].

In fact, some studies have suggested a trend toward thrombocytopenia during hemadsorption therapy [10, 18]. In this patient, preoperative platelet count was measured to be 74 × 10^9^/L and thus we felt that CytoSorb® therapy could be used safely. The expectation was that this patient would require platelet transfusion following the termination of cardiopulmonary bypass. Despite the presence of postoperative thrombocytopenia, postoperative platelet counts did not significantly differ from baseline. Therefore, we do not believe that CytoSorb® therapy significantly impacted platelet concentrations or perioperative transfusion requirements.

## Conclusion

Overall, we felt that CytoSorb® use during cardiopulmonary bypass was safe and feasible, and may have contributed to improved postoperative outcomes in our patient. Integration into the cardiopulmonary bypass circuit was uncomplicated and did not lead to any device-related complications. Future studies should aim to identify key patients in whom CytoSorb® hemoadsorption therapy would be beneficial. Hemoadsorption therapy may prove a promising therapeutic option in critically ill patients at risk of significant postoperative vasoplegia and multiorgan injury following prolonged and complex cardiac surgery.

## Data Availability

The datasets used and/or analyzed during the current study are available from the corresponding author on reasonable request.

## Abbreviations

CPB: Cardiopulmonary bypass
FDA: Food and Drug Administration
LVEF: Left ventricular ejection fraction
ACT: Activated clotting time
GFR: Glomerular filtration rate
pRBC: Packed red blood cells
